# Riboflavin as a Regulator of Cellular Metabolism: From the Classic Vitamin B2 to a Key Redox Factor

**DOI:** 10.3390/nu18193223

**Published:** 2026-09-29

**Authors:** Julia Kaźmierczak-Barańska, Krzysztof Halczuk, Bolesław T. Karwowski

**Affiliations:** DNA Damage Laboratory, Food Science Department, Faculty of Pharmacy, Medical University of Lodz, ul. Muszynskiego 1, 90-151 Lodz, Poland; krzysztof.halczuk@umed.lodz.pl (K.H.); boleslaw.karwowski@umed.lodz.pl (B.T.K.)

**Keywords:** riboflavin, prooxidation, antioxidation, human health

## Abstract

Riboflavin, i.e., vitamin B2, was first isolated by Kuhn and Győrgy in 1933, who reported that it acts as a growth factor. Riboflavin has long been known to play a key role in energy metabolism and cellular respiration, and its deficiency negatively affects skin and mucous membrane health. More recent studies have found it to regulate mitochondrial metabolism and to serve as the core of flavin coenzymes (FAD, FMN), which enable the respiratory chain and β-oxidation. Riboflavin also plays a key role as an antioxidant. While it is not a classic free antioxidant, it supports the enzymatic antioxidant system by supporting glutathione reductase, thioredoxin reductase, and ROS neutralization. On the other hand, when exposed to light, riboflavin exhibits photosensitizing properties that generate reactive oxygen species. As a result, it can initiate oxidative processes involving lipids, proteins, and nucleic acids, thereby exhibiting a prooxidative effect. The aim of this review is to provide an overview of the current understanding of the role of riboflavin in cellular and human health.

## 1. Introduction

**Figure 1:**
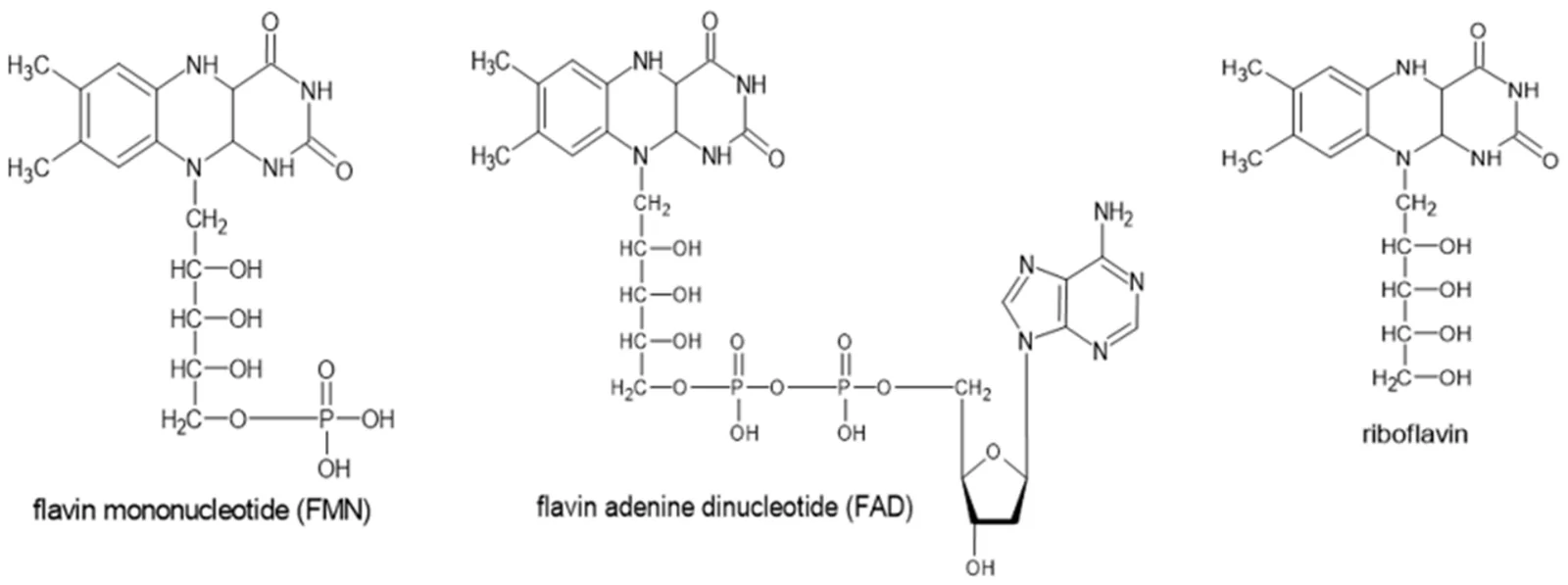
The structure of riboflavin, flavin mononucleotide (FMN), and flavin adenine dinucleotide (FAD).

Riboflavin is a water-soluble vitamin that occurs naturally in food. In addition to the free form, it is also found as two biologically active derivatives, *viz.* flavin adenine dinucleotide (FAD) and flavin mononucleotide (FMN) (Figure 1), which are bound to proteins to form flavoproteins [1]. In the digestive system, the flavoproteins release FMN and FAD, which are hydrolyzed to free riboflavin with the participation of alkaline phosphatases and FMN/FAD pyrophosphatase [2]. Only free riboflavin is absorbed in the intestines; after absorption, it is reconstituted into FMN, with the participation of riboflavin kinase, and subsequently into FAD with the participation of FAD synthase [3,4]. Flavins are chromophores involved in oxidation and reduction processes within cells.

Flavin coenzymes (FAD, FMN) act as electron carriers in oxidation and reduction reactions. They participate in energy-generating reactions from macronutrient molecules, which can also increase oxidative stress [5,6]. While riboflavin status is not routinely measured in healthy individuals, riboflavin deficiency can be effectively determined by measuring the erythrocyte glutathione reductase activity coefficient (EGRAC); this is achieved in vitro by determining the ratio of glutathione reductase activity in the presence of added FAD to its activity without added FAD [7,8]. A value of ≥1.30 generally indicates suboptimal riboflavin status, and ≥1.40 indicates deficiency [9]. Riboflavin status is also commonly determined by fluorometric measurement of 24 h urinary excretion; this can be expressed as the total amount of excreted riboflavin, or as the level relative to the amount of excreted creatinine [10].

As riboflavin is a photosensitive compound [11], its levels tend to decrease in people undergoing light therapy, e.g., for skin conditions [12,13]. More generally, a survey in Ireland found more than 50% of older adults to have low or deficient riboflavin status (i.e., EGRAC > 1.3), and approximately 20% to have an inadequate intake of riboflavin [14]. Clinical deficiency is characterized by mucocutaneous symptoms and anemia; severe or prolonged deficiency results in neurological and ophthalmological disorders, reduced physical performance, and developmental problems [8]. WHO/FAO [15] set a Recommended Nutrient Intake for men and women at 1.3 and 1.1 mg/day, respectively.

No examples of riboflavin overconsumption have been reported, as excess riboflavin is excreted, primarily in the urine [16]. Furthermore, the body is incapable of absorbing more than 27 mg of riboflavin at a time from the gastrointestinal tract [17].

Interestingly, recent studies indicate that riboflavin may play a role in the treatment of various neurological and oncological conditions. For example, riboflavin transporter deficiency (RTD), multiple acyl-CoA dehydrogenase deficiency (MADD), and migraine have been found to respond well to high-dose riboflavin supplementation (400 mg/day). Recent data also suggests that treatment may yield potential benefits for the treatment of Parkinson’s disease (90 mg/day), Alzheimer’s disease, multiple sclerosis (50 mg/day), and acute brain injury. As neurons are characterized by high oxygen consumption and lipid content, they are particularly susceptible to oxidative damage, and their health is heavily dependent on flavin coenzyme availability. Furthermore, FAD and FMN have been found to inhibit neuroinflammatory signaling [18,19].

In addition, cancer cells (melanoma, brain cancer, colorectal cancer) express high levels of riboflavin transporters, mainly RFVT1 and RFVT3 [20], possibly due to their higher energy requirement than normal cells, which would require more vitamin B2 derivatives [21]. In their study, the authors assessed gene and protein expression levels of RFVT in tumor tissue and the surrounding normal mucosa in 24 patients with CRC. The authors demonstrated that the expression level of the RFVT1 gene is significantly lower in tumor tissue compared to normal mucosa, whereas the expression levels of the RFVT2 and RFVT3 genes were significantly higher in tumor tissue. Based on these results, the authors hypothesized that RFVT3 is the primary factor responsible for the observed increase in intracellular flavin content [21]. These transporters could potentially be exploited for targeted drug delivery: attaching drug molecules or carriers to riboflavin may provide greater therapeutic precision, reduce damage to healthy tissues and minimize side effects [22]. However, the knowledge that the uptake of certain nutrients is increased by cancer cells may dangerously lead to the adoption of nutrient-restriction strategies. It is well known that supplementation with glutamine or taurine carries the potential risk of promoting cancer cell growth [23].

The role of riboflavin in cancer prevention and treatment is unclear, and epidemiological evidence, including meta-analyses, is contradictory and inconsistent. For example, Chen et al. indicate that riboflavin supplementation (72 h, 0.5 µM) does not promote cancer cell growth, indicating that dietary riboflavin supplementation is safe [24]. In vitro, studies using colorectal cancer (CRC) cells showed that elevated riboflavin concentrations (10 µM) did not result in increased cancer cell proliferation. In contrast, administering riboflavin-enriched drinking water (40 ng/mL) to mice with CRC significantly inhibited colorectal tumor growth, particularly at higher doses [24]. A retrospective case–control study in a Chinese population showed higher serum riboflavin concentrations to be associated with an increased risk of sporadic CRC [25]. Another recent study suggests that riboflavin may promote resistance to ferroptosis: it has been found to support the action of a key ferroptosis inhibitor (FSP1), which could preserve cell membrane integrity by inhibiting phospholipid peroxidation [26]. This ability to modulate ferroptosis via FSP1 suggests that riboflavin may play a central role in cancer cell viability by supporting cell membrane integrity during oxidative stress. The effect of riboflavin is indirect and depends on its ability to promote protection against lipid peroxidation via FSP1, rather than on any direct oncogenic effect of riboflavin itself (Table 1).

## 2. The Dual Nature of Riboflavin

**Figure 2:**
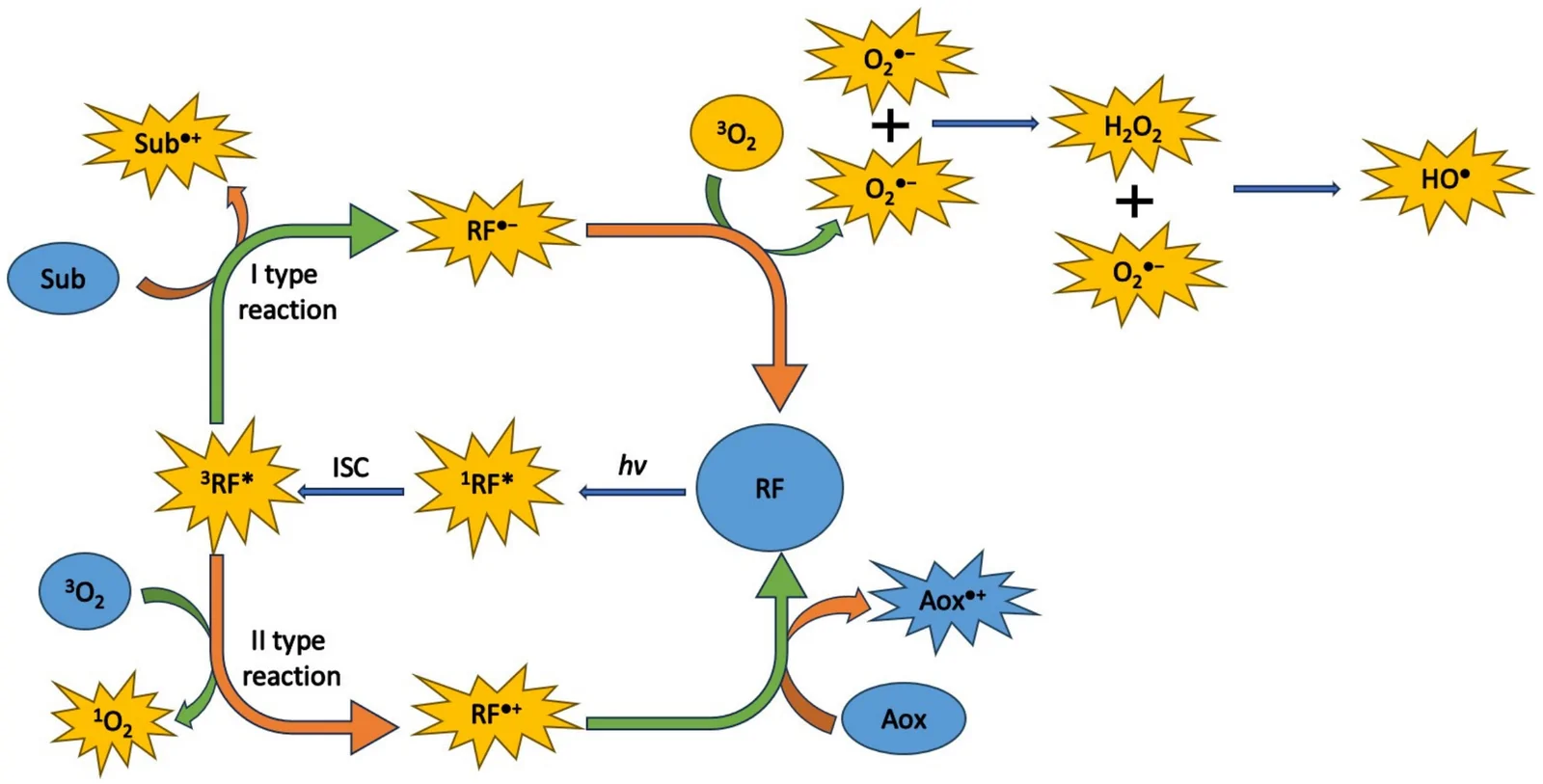
The excitation process of riboflavin and Type I and Type II photosensitization reactions [11,34]. Green arrows indicate oxidation reactions; orange arrows indicate reduction reactions. ISC—intersystem crossing, hv—light exposure. Under the influence of light, riboflavin forms a short-lived singlet excited state (^1^RF*) as an intermediate. Subsequently, a triplet state (^3^RF*) is formed because of an intersystem crossing. In a Type I reaction, an electron is transferred from ^3^RF* to a neutral organic substrate (Sub), such as a lipid or protein. This can cause irreversible damage to the substrate and results in the formation of a Sub cation radical and an RF anion radical (RF^●−^). RF^●−^ is quenched with the participation of oxygen to form O^2●−^, which can also damage neutral organic compounds. In a Type II reaction, ^3^RF* is quenched with the participation of ^3^O_2_, forming singlet oxygen and an RF cation radical (RF^●+^). Subsequently, RF^●+^ can be quenched with the participation of antioxidants (Aox).

Riboflavin is not a classic free antioxidant, but it performs antioxidant functions through enzymatic mechanisms. Intracellular riboflavin is phosphorylated to flavin mononucleotide (FMN) and then metabolized to flavin adenine dinucleotide (FAD) (Figure 2). Both FMN and FAD play a key role as cofactors in energy metabolism and are essential for numerous oxidation and reduction reactions in aerobic life forms. Their most important role is their involvement in oxidation and reduction processes: FMN acts as the primary electron acceptor in the first stage of the respiratory chain (electron transport chain), where it is reduced by the oxidation of NADH. Complex II (whose coenzyme is FAD) receives electrons from succinate, which reduces FAD to FADH_2_; these electrons are then transferred via FADH_2_ to ubiquinone. Riboflavin deficiency or defects in FAD and FMN can lead to disturbances in the respiratory chain, mitochondrial dysfunction, and increased ROS production, impairing cellular antioxidant mechanisms [28,29].

Depending on the biochemical context, redox conditions, and exposure to light, riboflavin can exhibit both antioxidant and pro-oxidant properties. While its antioxidant activity is well documented in enzymatic redox systems, its pro-oxidant activity is primarily observed during photosensitization, leading to the formation of reactive oxygen species (ROS).

### 2.1. Riboflavin Acts as a Photosensitizer and a Light-Activated Pro-Oxidant

Upon absorption of UV-VIS light, riboflavin transitions to an excited state. When excited, it can transfer energy, by generating singlet oxygen for example, or participate in oxidation and reduction reactions, leading to the photosensitization and oxidation of various compounds. Exposure to blue light or UVA radiation causes spin transitions to singlet excited states (^1^RF*), and a subsequent intersystem crossing results in the generation of an excited triplet state (^3^RF*). The triplet state is a relatively stable radical, with a lifetime on the order of microseconds, that demonstrates a high oxidizing potential (E0 ≈ +1.7 V relative to NHE) [11,30,31].

The triplet state is responsible for photosensitization, which can occur via two main pathways (Figure 2). In the first, a radical reaction can occur, in which the riboflavin triplet reacts with the substrate through electron or proton transfer, leading to the formation of radicals that are subsequently oxidized. In the second, energy can be transferred to molecular oxygen; in this case, the riboflavin triplet transfers energy to a molecular oxygen molecule, resulting in the formation of singlet oxygen, a highly reactive oxidizing agent.

As the riboflavin molecule exists as a conjugated aromatic system, i.e., an isoalloxazine ring, it strongly absorbs blue light, is easily excited (S1), and rapidly transitions to the triplet state [31,32]. When in this triplet state, flavins are excellent carriers of energy and electrons, and effective sensitizers of singlet oxygen; in particular, riboflavin can directly interact with, for example, deoxynucleotides and damage them. The isoalloxazine ring possesses numerous sites that may demonstrate activity in proton transfer, hydrogen atom addition, and electron exchange reactions (Figure 2). The triplet state can act as an oxidizing agent by accepting an electron from a donor (e.g., thiols, NADH), resulting in the formation of the semi-reduced form (the RF^•–^ anion radical). Conversely, it can act as a reducing agent by donating an electron to an acceptor (e.g., oxygen, Fe^3+^), resulting in the formation of the flavin cation radical (the semi-oxidized form). The RF^•–^ anion radical can reduce oxygen to form the superoxide anion radical O_2_^•–^, and can also react with biological substrates or undergo complete reduction to the RFH_2_ form. In contrast, the RF^•+^ cation radical, as a strong oxidizing agent, can damage amino acids, lipids, and thiols [11,33,34].

One promising avenue for the development of new breast cancer treatments concerns the administration of photosensitizing agents that selectively accumulate in cancer cells; these can then be “activated” by exposure to visible light [35]. In one study, riboflavin (0–100 μM) was found to exhibit no cytotoxicity toward non-cancerous cell lines (L929) or breast cancer cell lines (MCF-7, MDA-MB-231, and MDA-MB-468) when not exposed to visible light. However, when exposed to light (LED light (distance ∼10 cm, Lux = 5000) for 20 min, four hours after riboflavin treatment), the growth of breast cancer cell lines was significantly inhibited (IC50 values for riboflavin against MCF-7 were 22.5 μM, and against MDA-MB-231, 40.5 μM), in a dose-dependent manner, compared to the non-cancerous cell line; furthermore, riboflavin induced DNA damage and fragmentation in the cancer cells, as demonstrated by TUNEL (terminal deoxynucleotidyl transferase dUTP nick end labeling) assay, which enables the visualization and quantitative assessment of DNA fragmentation in cells undergoing apoptosis. The minimal effect on normal cells indicates that riboflavin photosensitivity treatment may have potential as a selective method to support breast cancer treatment.

However, without exposure to light, riboflavin undergoes only the classical redox cycle. It has been proposed that riboflavin can scavenge both ^●^OH and ^●^OOH radicals via the HAT (hydrogen atom transfer) mechanism [36]. The generalized quenching reaction can be written as follows:

R−OH+OH →R−O●+H2O●R−OH+OOH →R−O●+H2O2●

### 2.2. The Role of Riboflavin in Cellular Antioxidant Defense via Flavin-Dependent Redox Systems

**Figure 3:**
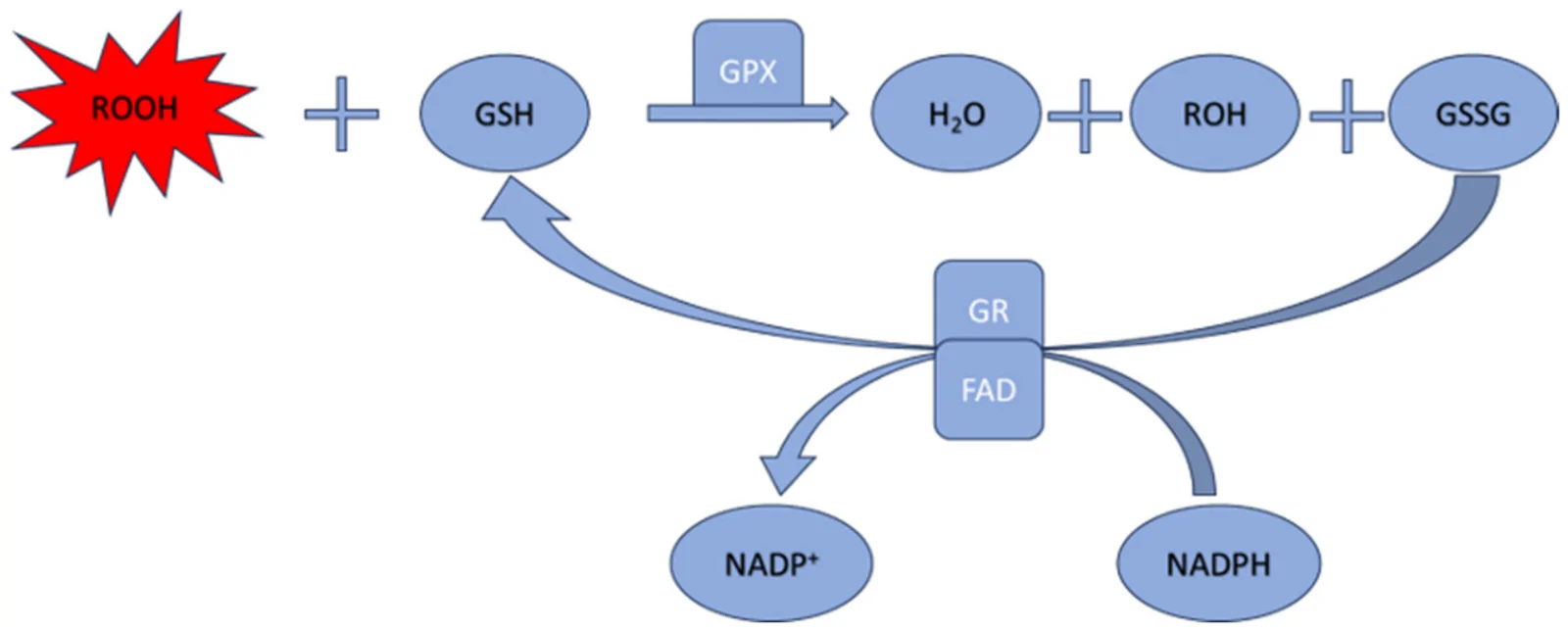
The role of FAD (the active form of vitamin B2) in glutathione regeneration and the protection of biomolecules. ROOH—peroxidized substrate (e.g., a lipid); GSH—glutathione; GPX—glutathione peroxidase; ROH—reduced substrate; GSSG—oxidized glutathione; GR—glutathione reductase; FAD—flavin adenine dinucleotide; NADPH/NADP^+^—nicotinamide adenine dinucleotide phosphate.

Riboflavin deficiency can worsen oxidative stress primarily because of a lack of FAD/FMN. In particular, FAD is needed for the operation of glutathione reductase, which regenerates the reduced form of glutathione (GSH), a key antioxidant needed to soak up free radicals in cells. In this regeneration process, FAD accepts electrons from NADPH and transfers them to the disulfide bridge in oxidized glutathione (GSSG), restoring it to its active form (Figure 3) [37].

Therefore, riboflavin deficiency leads to a reduction in glutathione reductase activity. The resulting increase in oxidative stress reduces glutathione levels and promotes the accumulation of free radicals and lipid peroxidation, as well as DNA and protein damage [5]. Riboflavin is also essential for mitochondrial energy metabolism (complexes I and II), and its deficiency disrupts the electron transport chain and increases superoxide levels [38]. Furthermore, riboflavin increases the expression of heme oxygenase (HO-1) and regulates mitochondrial biogenesis and oxidative stress through the AMPK/PGC-1α/HO-1 signaling pathway [39]. Deficiency also reduces antioxidant enzyme activity and increases MDA levels, a marker of lipid peroxidation. In contrast, elevated riboflavin/FMN/FAD levels correlate with increased SOD and CAT activity during adaptation to oxidative stress.

#### 2.2.1. The Importance of Riboflavin for Antioxidant Defense and Mitochondrial Function

Riboflavin transporter deficiency (RTD), also known as Brown–Vialetto–Van Laere syndrome, is a rare autosomal recessive disorder that affects motor neurons and limits riboflavin availability in cells [27,40]. The incidence is estimated to be less than 1 in 1,000,000 [41]. In young patients, the disease phenotype and prognosis depend on the timing of high-dose riboflavin (RF) treatment initiation (20–80 mg/kg/day for symptomatic cases and 5–10 mg/kg/day for presymptomatic cases) [27]. Riboflavin transporter deficiency is known to influence the morphology of mitochondria in motor neurons, with electron microscopy studies indicating swollen organelles with disrupted cristae [42]. Deficiency was also found to be associated with a shortening of neuronal processes, as indicated by confocal analyses, and significantly lower levels of SOD2 expression in the motor neurons. Combined treatment with riboflavin and *N*-acetylcysteine restored normal mitochondrial morphology, and statistical analysis revealed a significant (*p* ≤ 0.01) decrease in the number of damaged mitochondria in the neurons.

High doses of riboflavin have been shown to have a protective effect on the brain, which may be associated with reduced oxidative stress [43]. Rats were treated with riboflavin (100 mg/kg) during the neonatal period. Riboflavin treatment has been found to reduce the levels of protein carbonyl, an indicator of oxidative protein degradation, and malondialdehyde (MDA), a marker of lipid peroxidation, in the brains of rats. Riboflavin also improved the activity of superoxide dismutase (SOD) and glutathione-S-transferase (GST) [44] and enhanced the function of neuronal mitochondria.

Mitochondrial protection is an extremely important aspect of nervous system health. Mitochondria ensure the energy homeostasis needed for nervous system function and participate in the biosynthesis of lipids that form the myelin sheath. They also actively supply the ATP necessary for the transmission of nerve impulses. In addition, treatment with high doses of vitamin B2 increased the thickness of the myelin sheath in neurons of healthy rats [43]. Riboflavin treatment also improves synaptic communication in a healthy brain and reduces neuronal disorganization and degeneration in a stroke-affected brain.

Mitochondrial activity plays a crucial part in the development of cancer and its associated physical wasting. Cancer cells are characterized by an impaired energy metabolism and cancer-related cachexia: the cells exhibit lower net ATP production despite a high rate of glycolysis [45]. In a study of mitochondria of the mammary gland, the enzymes of the Krebs cycle (isocitrate dehydrogenase, α-ketoglutarate dehydrogenase, succinate dehydrogenase, and malate dehydrogenase) and oxidative phosphorylation (NADH dehydrogenase and cytochrome c oxidase) exhibited significantly lower activity in rats with breast cancer compared to healthy controls (*p* < 0.05) [46]. Mammary carcinoma (Group II) was induced with 25 mg/mL DMBA. The activity of these enzymes was significantly restored to near-normal levels following treatment with combination therapy consisting of 45 mg/kg body weight (per day) of riboflavin together with niacin (100 mg/kg body weight per d) and coenzyme Q (40 mg/kg body weight per d). Nutritional therapy is a key component of treatment for cancer-related cachexia and can help control the course of the disease.

Riboflavin has been identified as an inhibitor of inflammatory proteasomes. It has the potential to suppress the production of inflammatory mediators in aging organisms and indirectly reduce the risk of age-related diseases [47]. Qureshi et al. observed that 40 µM riboflavin inhibited LPS-induced TNF-α secretion in macrophages by 68%, proteasomal degradation of IkB by 50%, and NF-κB activation by 34%; it also prevented the migration of NF-κB into the nucleus, and its binding to the promoter regions of various pro-inflammatory genes.

The processes modulating inflammation also play an important role in the immune system. For example, ROS produced by specialized cells act as defensive and signaling molecules associated with innate immunity. In peritoneal macrophages infected with *S. aureus*, pretreatment with riboflavin was found to enhance their phagocytic activity, support antibiotic action, and promote the production of H_2_O_2_ and ROS [48]. Riboflavin enhances the endogenous antioxidant potential of macrophages, as shown by increased GSH levels and SOD activity, both alone and with antibiotics. It also inhibits bacterial catalase activity and reduces the intracellular survival of *S. aureus*, especially when given in combination with antibiotics.

#### 2.2.2. The Potential Role of Riboflavin in Reducing the Risk of Diabetes by Modulating Oxidative Stress

The antioxidant effects of riboflavin have been confirmed in humans in randomized, controlled clinical trials measuring redox biomarkers, glutathione metabolism, and the reduction of oxidative damage, both as monotherapy and as part of a multi-component diet. One large prospective cohort study in China assessed the risk of gestational diabetes in relation to riboflavin intake among 3036 women in their first and second trimesters of pregnancy [49]. Total riboflavin intake, comprising mean daily dietary intake and supplement use, was assessed using a modified version of the semi-quantitative Food Frequency Questionnaire (FFQ). It was found that total riboflavin intake was significantly and negatively associated with the risk of GDM. In the first trimester, riboflavin supplementation reduced the incidence of gestational diabetes by 42% in the fully adjusted model. Those with higher total riboflavin intake during the first and second trimesters had a lower risk of gestational diabetes than those with lower total riboflavin intake, after adjusting for potential confounding factors.

Riboflavin may protect tissues from oxidative damage resulting from increased free radical production and impaired antioxidant mechanisms, and this damage may play a part in the etiopathology of diabetes [50,51]. Studies in diabetic mice found that riboflavin treatment (10 and 20 mg/kg/day for 28 days) reduced lipid and protein peroxidation and increased the activity of antioxidant enzymes (GR) by 34% in the liver, 35% in the kidneys, and 52% in the pancreas [52]. Furthermore, the treatment restored the expression of GLUT-4 transporters in adipocytes and skeletal muscle, alleviated diabetes-induced histopathological changes in the liver and kidneys, and reduced DNA damage. This study illustrates the potential mechanistic role of riboflavin in modulating oxidative stress in the treatment of diabetes, rather than demonstrating its clinical efficacy. Hence, further research into the role of riboflavin in the treatment and prevention of diabetes is merited.

The effect of a combination of riboflavin with other antioxidant micronutrients (800 mg of dl-α-tocopherol acetate, 24 mg of β-carotene, 1.0 g of vitamin C, 200 μg of L-selenomethionine, 7.2 mg of riboflavin, 80 mg of niacin, 60 mg of zinc, and 5 mg of manganese) on patients (*n* = 47) with a history of sporadic colorectal adenoma was evaluated in a pilot, randomized, double-blind, placebo-controlled clinical trial [53]. After daily administration of the mixture for four months, significant reductions in the levels of the circulating inflammatory marker TNF-α (baseline: 3.01 pg/mL; after 4 months: 2.77 pg/mL) and the oxidative stress marker cystine (CySS) (37.1 µmol/L/mL; after 4 months 25.8 µmol/L/mL) were observed, particularly in nonsmokers. The mean plasma concentration of TNF-α decreased by 36% in the treatment group compared with the placebo group (*p* = 0.001), and the CySS concentration decreased by 39% (*p* = 0.02). However, these results should not be interpreted as evidence of riboflavin’s independent action; nevertheless, studies on dietary interventions often use multivitamin formulations, which reflect the biological reality, where antioxidant defense systems function through networks of interacting nutrients rather than isolated compounds.

#### 2.2.3. Clinical Assessment of the Effects of Riboflavin Supplementation on Antioxidant Status

In a prospective clinical intervention study (RISE-UP), 70 patients with Crohn’s disease (CD) of varying severity received 100 mg of riboflavin daily for three weeks [54]. The study examined the effect of riboflavin on oxidative stress, markers of inflammation, clinical symptoms, and the fecal microbiome. After three weeks of riboflavin supplementation, serum pro-inflammatory cytokine IL-2 levels were found to decrease while plasma free thiol levels increased, indicating a reduction in systemic oxidative stress. While riboflavin supplementation for 3 weeks improved the systemic redox state, analysis using shotgun metagenomic sequencing (MGS) showed no effect on the diversity, taxonomy, or metabolic pathways of the fecal microbiome. Reducing inflammation is an important therapeutic goal for improving the quality of life of patients with CD, and riboflavin, as a safe agent with anti-inflammatory potential, may serve as a supportive therapeutic tool.

Chronic physical exertion is associated with increased production of ROS and oxidative stress, and changes in serum antioxidant levels. Promising results were reported following a study of riboflavin in volunteer construction workers: one group consumed juice fortified with 1.3 mg of riboflavin (Group A) and a second consumed only apple juice (Group B) [55]. After 28 days, participants in Group (A) showed a significant increase in basal metabolic rate, i.e., a greater number of calories burned at rest. They also demonstrated improved total individual strength score (65.32), as well as improvements on the subjective fatigue and physical activity subscales of the fatigue severity scale (a 68.13% reduction in fatigue). Biochemically, they exhibited lower levels of MDA, an indicator of oxidative stress and lipid peroxidation (53.78% decrease), and higher catalase enzyme activity (54.65% improvement), and total serum antioxidant capacity (74.19% better). The results suggest that supplementing the diet with vitamin B2 can effectively reduce fatigue and exhaustion in active and/or physically active individuals.

Studies on diet and nutrition in the United Kingdom have found marginal riboflavin deficiency to be widespread among adolescent girls and young women [56]. Research has found 95% of girls to have poor riboflavin status, with an erythrocyte glutathione reductase activation coefficient (EGRAC) test result greater than 1.40 [57]. Supplementation with 2 and 4 mg/day for 8 wk. of riboflavin resulted in a significant improvement in riboflavin status, as indicated by a dose-dependent reduction in EGRAC. Changes in riboflavin status measures after 8 weeks of treatment for EGRAC were: −0.25 at 2 mg; and −0.37 at 4 mg of riboflavin. Subsequently, the improvement in riboflavin status led to an increase in circulating red blood cell count and hemoglobin concentration. In addition, a poorer riboflavin status at the start of the study was associated with a greater increase in red blood cell count or hemoglobin concentration: women in the highest EGRAC tertile at the start of the study (>1.65) showed a significantly greater increase in hemoglobin and red blood cell counts. The results of the RIBOFEM study [56] indicate that improving riboflavin status had a beneficial effect on certain hematological parameters in young women, and suggest that poor riboflavin status in women may disrupt iron metabolism. However, riboflavin status is rarely assessed, and vitamin B2 is rarely recognized as a vitamin that can be deficient.

This raises the question of whether supplementation in healthy individuals could have a beneficial effect on health: it is possible that riboflavin could increase extracellular antioxidant potential, which would serve as a preventive measure against oxidative stress. To address this question, the RIBOGUT study, a randomized, double-blind, placebo-controlled intervention trial, examined the effect of riboflavin supplementation on the composition of the gut microbiota in healthy individuals [58]. Importantly, no adverse effects were associated with the supplementation (50 or 100 mg of riboflavin for 14 days). However, no significant changes in albumin-corrected free thiol levels were observed following riboflavin supplementation, nor any other differences compared to the placebo group. Hence, supplementation did not result in any clear improvement in systemic redox status in healthy individuals.

In contrast, a pilot double-blind, randomized clinical trial with 30 patients found that continuous supplementation may be beneficial. A daily B-complex vitamin supplement resulted in reduced homocysteine levels and elevated serum TAC (Total Antioxidant Capacity) at the end of supplementation, and a decrease in TAC during the washout period. The supplement was administered daily for six weeks at doses approximately 2.5 times higher than the recommended daily intake [59]. However, TAC is primarily a biochemical marker of antioxidant status, not a direct measure of clinical benefit.

## 3. Conclusions

Due to its unique chemical structure, riboflavin can exhibit a certain degree of redox duality. While it may act as a pro-oxidant at very high doses and during intense exposure to light, it primarily exhibits antioxidant potential. Riboflavin also appears to play a key role in mitochondrial health by indirectly facilitating the respiratory chain through FAD and FMN. Healthy mitochondria maintain a balanced oxidant status, and ensure the proper course of energy reactions within the cell: the latter is needed to facilitate overall health and has been linked to obesity and the functioning of *inter alia* the pancreas, heart, and brain. Despite promising results that suggest riboflavin has multifaceted biological effects, the available scientific evidence remains limited due to significant heterogeneity in research models, doses used, and experimental designs. Nevertheless, there remains a need for well-designed, randomized, large-scale clinical trials to better establish the role of riboflavin supplementation in metabolic conditions such as diabetes, CVD and neurodegenerative diseases, and among healthy individuals.

## Figures and Tables

**Table 1:** A summary of current knowledge of riboflavin (vitamin B2): its properties, deficiency/excess implications, and guidance across special populations.

	Current Knowledge
Properties of Riboflavin	Daily Requirements: ~1.1 mg/day for women, ~1.3 mg/day for men; Pregnant women riboflavin intake up to 1.4 mg/d [15]
	Food Sources: Offal (especially liver), whole grains, dairy products (milk, yogurt, cheese), green leafy vegetables. It is widely used as a food coloring (E 101) due to its intense yellow color [14]
	Stability: Light-sensitive, thermally stable [11,13]
Excess	High-Dose Benefits: migraines (400 mg/day), neurological disorders Parkinson’s disease (90 mg/day), multiple sclerosis (50 mg/day) [20]
	Toxicity: Riboflavin is water soluble, so excess amounts are excreted in the urine. There is no evidence of toxicity when consumed through diet. High-dose supplementation results in bright yellow urine (harmless) [16]
Specific Patient/ Populations	Pregnancy/Lactation: Increased requirement [8]
	Vegetarian athletes: The requirement increases with a higher metabolic rate (e.g., athletes, breastfeeding women) [10]
	Riboflavin transporter deficiency: Neurological disorder: Brown–Vialetto–Van Laere or Fazio–Londe syndrome (associated with mutations in riboflavin transporter gene: SLC52A3, which encodes the intestinal riboflavin transporter RFVT3) [27]

## Data Availability

No new data were created or analyzed in this study. Data sharing is not applicable to this article.

## Abbreviations

The following abbreviations are used in this manuscript:

CRC	Colorectal Cancer
CVD	Cardiovascular Disease
CySS	Cystine, Marker of Oxidative Stress
EGRAC	Erythrocyte Glutathione Reductase Activation Coefficient
FAD	Flavin Adenine Dinucleotide
FFQ	Food Frequency Questionnaire
FMN	Flavin Mononucleotide
GSH	Glutathione
GST	Glutathione S-Transferases
LPS	Lipopolysaccharides
MDA	Malondialdehyde
MADD	Multiple Acyl-Coa Dehydrogenase Deficiency
NADP	Nicotinamide Adenine Dinucleotide Phosphate
RF	Riboflavin
ROS	Reactive Oxygen Species
RTD	Riboflavin Transporter Deficiency
SOD	Superoxide Dismutase
TAC	Total Antioxidant Capacity
TNF	Tumor Necrosis Factor
